# The Mediating Role of Psychological Capital in the Relationship Between Occupational Stress and Fatigue: A Cross-Sectional Study Among 1,104 Chinese Physicians

**DOI:** 10.3389/fpubh.2020.00012

**Published:** 2020-02-28

**Authors:** Fangqiong Tian, Qianyi Shu, Qi Cui, Lulu Wang, Chunli Liu, Hui Wu

**Affiliations:** ^1^Department of Social Medicine, School of Public Health, China Medical University, Shenyang, China; ^2^Party Committee Office, Shengjing Hospital of China Medical University, Shenyang, China; ^3^Library of China Medical University, Shenyang, China

**Keywords:** fatigue, occupational stress, psychological capital, mediating effect, physician

## Abstract

**Purpose:** This study aimed to explore the association of occupational stress with fatigue and to examine the mediating role of psychological capital (PsyCap) among Chinese physicians.

**Materials and Methods:** A cross-sectional study was conducted in Liaoning province, China, in 2018. Using a multistage stratified sampling method, a total of 1,500 physicians participated and 1,104 (73.6%) physicians responded effectively. The study used a self-administered questionnaire consisting of the 14-item Fatigue Scale (FS-14), the Effort-reward Imbalance questionnaire (ERI), the Psychological Capital Questionnaire (PCQ) and items about demographic and working characteristics. Hierarchical multiple regression analyses were performed to explore the association of occupational stress, PsyCap, and fatigue among physicians. Asymptotic and resampling strategies were used to examine the mediating effect.

**Results:** The incidence of fatigue among Chinese physicians was 83.70%. The average level of fatigue was 7.96 ± 3.95 (mean ± SD). Occupational stress and PsyCap were significantly associated with fatigue. PsyCap significantly mediated the association of ERR (*a* × *b* = 0.106, bias-corrected and accelerated 95% confidence interval [BCa 95% CI]: 0.078, 0.138) and overcommitment (*a* × *b* = 0.068, BCa 95% CI: 0.044, 0.092) with fatigue. Two important components of PsyCap, self-efficacy and resilience, play more important roles in the mediating effect.

**Conclusions:** The level of fatigue among Chinese physicians was high, which should be taken seriously by management. PsyCap could mediate the association between occupational stress and fatigue. The intervention strategies and measures to relieve fatigue could be focused on physicians' positive PsyCap improvement.

## Introduction

Fatigue is expressed as a condition characterized by physical or mental exhaustion after prolonged periods of exertion without adequate rest and recovery (1). According to a survey among general working population in 2018, the rates of fatigue for sedentary workers and physical workers were 17.0 and 38.9%, respectively (2). In reality, fatigue is a longstanding problem within the health-care occupations in many countries, such as China, the United States, the United Kingdom, and Australia, and especially among physicians (3–7). O'Donnell's research suggested that nearly half of physicians (45.4%) are extremely fatigued (6). Physicians are more vulnerable to fatigue than are those in other occupational groups (8). Long-term fatigue of physicians could lead to adverse health consequences, like musculoskeletal disorders, poor mental health status, and increased error and accident at work (9, 10). It means fatigue has a major impact not only on physicians' life quality and work efficiency but also on patients' safety and satisfaction with health-care services. Therefore, more attention should be paid on the influencing factors and the formation mechanism of fatigue in order to develop targeted preventive measures against fatigue among physicians.

Demographics and working characteristics are two considerable factors affecting fatigue (11). It is reported that fatigue could be influenced by age, marital status, and educational level (3). Another study found the relation between fatigue and poorly designed shift rotas among junior physicians. Particularly, shift work and night duty predispose physicians to a high level of fatigue (12). In addition, fatigue is a multifactorial phenomenon that can be influenced by psychosocial factors. For instance, it is reported that fatigue was related with occupational stress and psychological distress among bus drivers and industrial workers (13, 14). Fatigue and occupational stress also showed a significantly positive correlation among nurses (15, 16).

Occupational stress usually refers to the physical and mental health pressures of employees, as well as physical disturbances caused by imbalances between employee's capabilities and objective needs (17). As two of the leading occupational stress models, effort–reward imbalance (ERI) model and job demand–control–support (DCS) model are extensively applied, but the ERI model appeared to be more predictive than the DCS model in Chinese workers (18). Siegrist's ERI model measures the balance of exertions and gains at work, which includes items on reward and intrinsic and extrinsic effort (19). One of the most important factors in ERI is effort–reward ratio (ERR), which evaluates whether the rewards are equal to the extrinsic effort. Overcommitment is the other component of ERI model, which is the individual's internal drive to achieve the goal, including the degree of physical and mental investment (20). Excessive commitment or imbalance between efforts and rewards would lead to occupational stress, which could trigger psychosomatic reaction and cause or exacerbate fatigue (21, 22). Therefore, occupational stress might significantly have an impact on fatigue among Chinese physicians.

Owing to the emergence of positive psychology in recent decades, scholars began to explore solutions to undesirable employee attitudes (fatigue, anxiety, and depression) from the perspective of psychological resources (23, 24). It is noticeable that internal psychological constructs, like self-efficacy, resilience, hope, and optimism, have played positive roles in relieving fatigue symptoms (25). Psychological capital (PsyCap) is an important positive psychological resource of individuals, defined by Luthans as “a positive psychological state that an individual performs in the process of growth and development” (26). PsyCap consists of four components of mental resource—self-efficacy, resilience, hope, and optimism—all of which can be measured and developed (27). PsyCap and its four components may be key to better understand the variation in stress, as well as intentions to negative behaviors (28). Previous studies have showed the a negative relation between PsyCap and fatigue in a variety of professions (27, 29). Analogously, Kim and Jang reported that seafarers' self-efficacy will have a negative effect on fatigue (30). Resilience also showed significant effects on fatigue among hospital employees (31). Therefore, PsyCap might be one of the keys to effectively prevent and relieve fatigue of Chinese physicians.

What is more, studies have indicated that occupational stress can directly affect psychological well-being and also can indirectly affect employee's attitude and health (32, 33). When occupational stress arises, PsyCap could psychologically buffer the effect of occupational stress on adverse outcomes (34) and bring more energy physically to the individual (35). It is well established that PsyCap can significantly affect the association between occupational stress with job burnout, depression, turnover intention, and job satisfaction among various occupational groups (36–39). For example, previous studies reported that PsyCap mediated the association of occupational stress (ERI) and burnout among bank employees and manufacturing workers (33, 36). However, whether PsyCap mediates the association between occupational stress and fatigue among physicians has not yet been determined. Therefore, it is necessary to find out the association between occupational stress, PsyCap, and fatigue among Chinese physicians.

On the basis of the above, we made the following three assumptions among Chinese physicians: (1) demographic and working characteristics such as age, gender, educational level, marital status, job rank, shift patterns, and night duty may be significantly associated with fatigue among physicians; (2) after demographic working characteristics were adjusted for, occupational stress would still be an important factor affecting fatigue; and (3) PsyCap could mediate the association between occupational stress and fatigue.

## Materials and Methods

### Study Design and Data Collection

A cross-sectional survey was conducted in Liaoning province, China, in 2018. With the use of a multistage stratified sampling method, a total of 10 public tertiary hospitals and 1,500 physicians were randomly selected. Liaoning province consists of 14 prefecture-level cities, which can be divided into five regions by geographical location: eastern, western, southern, northern, and central. In each geographic region, one city was randomly selected. In the second sampling stage, we sampled according to the proportion of hospitals in each city. Three tertiary public hospitals were randomly selected if the sampling city was a capital city; two tertiary public hospitals were randomly selected from the smaller cities. Particularly, one hospital was selected if the selected city only has one tertiary public hospital. In this study, 10 tertiary public hospitals were selected from five cities. In each selected hospital, 150 physicians were randomly selected by the random number table. There was no incentive for participating in this study. The self-administered questionnaires were distributed to 1,500 physicians after obtaining written informed consent. For each physician, it took 8–10 min to complete the questionnaire. Of all subjects, 1,104 physicians answered all items and scales completely, with an effective response rate of 73.6% eventually.

### Demographic Variables and Working Characteristics

We used a self-designed questionnaire to collect demographics and working characteristics including gender, age (years), educational level, marital status, job rank, shift patterns, and night duty. Age was categorized as “ <30,” “30–40,” and “>40.” Educational level was categorized as “junior college or lower,” “college,” and “graduate or higher.” Marital status was categorized as “single/widowed/divorced/separated” and “married/cohabiting.” Job rank was divided into “staff” and “director or deputy director.” Shift patterns were divided into “shift” and “fixed.” Night duty was defined as “yes” or “no.” All items were self-evaluated.

### Measurement of Fatigue

Fatigue was measured by the 14-item Fatigue Scale (FS-14), developed in 1992 by Chalder et al., which has been used to assess fatigue severity in China (40, 41). The score for each item is measured by a fatigue-related problem scored with two responses: 0 (no fatigue-related problem) and 1 (have fatigue-related problem). The total fatigue score was calculated ranging from 0 to 14. The Chinese version of the FS-14 has been used in physician groups in China, and it has adequate reliability and validity (42, 43). In our study, the Cronbach alpha coefficient of the FS-14 was 0.844.

### Measurement of Occupational Stress

Occupational stress was assessed using Siegrist's Effort–reward Imbalance questionnaire (ERI) (44). The Chinese version of the ERI scale was translated and provided by Li et al. (45). The questionnaire comprises 23 items and three subscales: extrinsic effort (6 items), reward (11 items), and overcommitment (6 items). For the ERI scale, occupational stress can be expressed by the ERR and overcommitment. The score of each response for extrinsic effort and reward is from 1 (not stressful) to 5 (very stressful). The ERR score was calculated by the following equation: ERR = 11 × effort/6 × reward. Responses for overcommitment are scored from 1 (strong disagreement) to 4 (strong agreement). The Chinese version of the ERI has been widely used in Chinese occupational groups and has been found to have good reliability and validity (32, 33). In our study, the Cronbach alphas for the extrinsic effort, reward, and overcommitment subscales and the total scale were 0.894, 0.955, and 0.877 and 0.776, respectively.

### Measurement of Psychological Capital

The 24-item Psychological Capital Questionnaire (PCQ) was used to measure PsyCap (46). The score for each of the four components of PsyCap (self-efficacy, hope, resilience, and optimism) is measured by six items scored from 1 (indicates strong disagreement) to 6 (indicates strong agreement). Higher values indicate a higher level of PsyCap and its components. The Chinese version of the PCQ has been proved to have satisfactory reliability and validity (47, 48). In our study, the Cronbach alpha coefficients for self-efficacy, hope, resilience, and optimism subscales and the total scale were 0.943, 0.952, 0.937, and 0.921 and 0.977, respectively.

### Statistical Analysis

**Figure 1:**
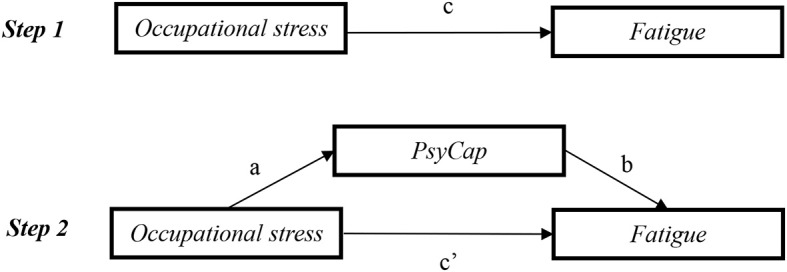
Mediating role of PsyCap in the association between occupational stress and fatigue. c, association of occupational stress with fatigue; a, association of occupational stress with PsyCap; b, association between PsyCap and fatigue after controlling for the covariates; c′ , association of occupational stress with fatigue after adding PsyCap as a mediator. PsyCap, psychological capital.

All statistical analyses were carried out using IBM SPSS Statistics 21.0 (IBM, Asia Analytics Shanghai), with two-tailed probability value of <0.05 considered to be statistically significant. Descriptive statistics for demographic and working characteristics of the physicians were showed with mean, standard deviation (SD), number (*n*), and percentage. The independent samples *t*-test and one-way (ANOVA) analysis were carried out to compare the difference of fatigue according to demographic and working characteristics of the participants. Pearson's correlation analysis was used for the correlation between fatigue, PsyCap, and occupational stress. Hierarchical multiple regression analyses were applied to investigate the factors in relation to fatigue. In hierarchical multiple regression analyses, variance inflation factor (VIF) values of all predictive variables <10, which indicated that collinearity was a negligible problem in the study. The variables in the models were centralized before regression analysis. In block 1 of the analysis, demographics and working characteristics were associated with fatigue in the univariate analysis (*P* < 0.05), and age and gender were entered as control variables. Occupational stress and PsyCap were entered into block 2 and block 3 as an independent variable and a mediating variable, respectively. The conceptual framework of this study is shown in Figure 1; in step 1, the aim was to verify the direct effect of occupational stress on fatigue (the *c* path) after adjusting covariates; in step 2, the aim was to verify the mediating effect of PsyCap. The asymptotic and resampling strategies developed by Preacher and Hayes were carried out to verify PsyCap as a potential mediator on the association between occupational stress and fatigue (49). The bootstrap estimate was based on 5,000 bootstrap samples. A bias-corrected and accelerated 95% confidence interval (BCa 95% CI) was calculated for each *a* × *b* product, and a BCa 95% CI excluding 0 significantly manifested mediation.

## Results

### Demographic and Working Characteristics of Subjects

The prevalence of fatigue among Chinese physicians was 83.70% (924). Demographic and working characteristics among physicians and group comparisons on fatigue are displayed in Table 1. The participants were in the average age of 37.92 ± 9.19, and the average fatigue score of the participants was 7.96 ± 3.95. There was a significant difference in the level of fatigue on age, educational level, and marital status. The score of fatigue in the age group of >40 years was significantly higher than that of other age groups (*P* < 0.05). In comparison with the high educational level physicians, low educational level physicians had significantly higher levels of fatigue (*P* < 0.01). The score of fatigue with a married or cohabiting status was significantly higher than that of participants who are single, divorced, widowed, or separated (*P* < 0.01). However, no significant differences in fatigue were observed among male and female physicians. In terms of working characteristics, physicians on shift reported a higher fatigue score than did those on fixed work (*P* < 0.01). Nevertheless, job rank and night duty were not significantly related to the fatigue of physicians.

**Table 1:** Demographic and working characteristics of subjects (*N* = 1,104) and comparisons on fatigues.

**Variables**	***n* (%)**	**Mean ±*SD***	***t*/*F* value**	***P*-value**
Age (years)			4.318	0.014
<30	268 (24.3%)	7.46 ± 3.55		
30–40	390 (35.3%)	7.88 ± 4.13		
>40	446 (40.4%)	8.34 ± 4.00		
Gender			0.605	0.437
Male	578 (52.4%)	8.05 ± 4.04		
Female	526 (47.6%)	7.86 ± 3.86		
Educational level			4.980	0.007
Junior college or lower	140 (12.7%)	8.67 ± 4.56		
College	605 (54.8%)	8.07 ± 3.74		
Graduate or higher	359 (32.5%)	7.50 ± 4.00		
Marital status			6.915	0.009
Single/widowed/ divorced/separated	388 (35.1%)	8.38 ± 4.13		
Married/cohabiting	716 (64.9%)	7.73 ± 3.84		
Job rank			0.403	0.526
Staff	905 (82.0%)	8.00 ± 3.92		
Director or deputy director	199 (18.0%)	7.80 ± 4.10		
Shift patterns			9.121	0.003
Shift	717 (64.9%)	8.22 ± 3.70		
Fixed	387 (35.1%)	7.47 ± 4.34		
Night duty			3.203	0.074
Yes	844 (76.4%)	8.08 ± 3.93		
No	260 (23.6%)	7.58 ± 4.02		

### Correlations Among Occupational Stress, Psychological Capital, and Fatigue

The results of the correlation analysis among occupational stress, PsyCap, and fatigue are shown in Table 2. Age, occupational stress (ERR and overcommitment), PsyCap, and the four components of PsyCap were all significantly correlated with fatigue. ERR and overcommitment were positively correlated with fatigue and negatively correlated with PsyCap. Self-efficacy, resilience, hope, optimism, and PsyCap were negatively correlated with fatigue.

**Table 2:** Pearson's correlation coefficients between study variables.

**Variables**	**Mean ± SD**	**1**	**2**	**3**	**4**	**5**	**6**	**7**	**8**
1. Age	37.92 ± 9.19	1							
2. ERR	1.24 ± 0.70	0.125^**^	1						
3. Overcommitment	15.37 ± 3.69	−0.034	0.219^**^	1					
4. Self-efficacy	22.46 ± 6.80	−0.101^**^	−0.282^**^	−0.171^**^	1				
5. Resilience	21.22 ± 7.04	−0.049	−0.335^**^	−0.236^**^	0.790^**^	1			
6. Hope	21.18 ± 7.12	−0.083^**^	−0.297^**^	−0.186^**^	0.837^**^	0.800^**^	1		
7. Optimism	21.58 ± 6.31	−0.055	−0.341^**^	−0.231^**^	0.785^**^	0.883^**^	0.793^**^	1	
8. PsyCap	87.21 ± 28.05	−0.086^**^	−0.340^**^	−0.236^**^	0.885^**^	0.931^**^	0.895^**^	0.918^**^	1
9. Fatigue	7.96 ± 3.95	0.067^*^	0.416^**^	0.201^**^	−0.465^**^	−0.460^**^	−0.450^**^	−0.447^**^	−0.410^**^

* P < 0.05,

** *P < 0.01 (two-tailed)*.

*ERR, effort–reward ratio; PsyCap, psychological capital*.

### Mediating Role of Psychological Capital in the Association Between Occupational Stress and Fatigue

As shown in Table 3, the hierarchical multiple regression analyses were conducted to explore the contributing and mediating factors associated with fatigue. First of all, the VIFs of all independent variables were less than 10, which means that collinearity did not mislead in the estimate. After age, gender, educational level, marital status, and shift patterns were adjusted for, the EER was positively associated with fatigue (β = 0.368, *P* < 0.01), and overcommitment was positively associated with fatigue (β = 0.144, *P* < 0.01). Two components of occupational stress accounted for 17.7% of the variance in block 2. In block 3, PsyCap (β = –0.379, *P* < 0.01), self-efficacy (β = –0.203, *P* < 0.01), and resilience (β = −0.120, *P* < 0.05) were negatively associated with fatigue, whereas hope and optimism were not significantly associated with fatigue. The four components of PsyCap accounted for an additional 12.6% of the variance in model 2 of block 3. In block 3, the standardized regression coefficient (β) of ERR and overcommitment was both reduced. Thus, PsyCap, self-efficacy, and resilience could probably become mediators in the association between occupational stress and fatigue.

**Table 3:** Hierarchical multiple regression analysis of the association of occupational stress and PsyCap with fatigue.

**Variables**	**Block 1 (*******β*******)**		**Block 2 (*******β*******)**		**Block 3 (*******β*******)**			
					**Model 1**		**Model 2**	
	**β**	**VIF**	**β**	**VIF**	**β**	**VIF**	**β**	**VIF**
Age	0.078^*^	1.076	0.037	1.102	0.017	1.105	0.014	1.111
Gender	−0.001	1.039	0.013	1.044	0.024	1.045	0.024	1.047
Educational level	−0.072^*^	1.051	−0.057^*^	1.064	−0.028	1.071	−0.021	1.087
Marital status	−0.073^*^	1.127	−0.068^*^	1.149	−0.050	1.152	−0.055^*^	1.159
Shift patterns	0.072^*^	1.057	0.071^*^	1.070	0.069^**^	1.070	0.064^*^	1.077
EER			0.368^**^	1.103	0.262^**^	1.196	0.267^**^	1.208
Overcommitment			0.144^**^	1.110	0.077^**^	1.147	0.080^**^	1.152
PsyCap					−0.379^**^	1.175		
Self-efficacy							−0.203^**^	4.095
Resilience							−0.120^*^	5.406
Hope							−0.081	4.187
Optimism							−0.004	5.248
*F*	5.445^**^		121.629^**^		197.741^**^		50.993^**^	
Adjusted *R^2^*	0.020		0.196		0.319		0.320	
Δ*R^2^*	0.024		0.177		0.122		0.126	

* P < 0.05,

** *P < 0.01 (two-tailed). Age was handled as a continuous variable. Gender, male vs. female. Marital status, single/widowed/divorced/separated vs. married/cohabiting. Shift patterns, shift vs. fixed*.

*EER, effort–reward ratio; PsyCap, psychological capital; VIF, variance inflation factor*.

After PsyCap's mediating effect was tentatively explored by hierarchical regression analysis, asymptotic and resampling strategies were used to examine the mediating roles of PsyCap. As shown in Table 4, PsyCap (*a* × *b* = 0.106, BCa 95% CI: 0.078, 0.138), self-efficacy (*a* × *b* = 0.046, BCa 95% CI: 0.016, 0.011), and resilience (*a* × *b* = 0.034, BCa 95% CI: 0.009, 0.070) significantly mediated the association between EER and fatigue, and the mediating effect of self-efficacy and resilience accounted for 17.20% and 12.71%, respectively. PsyCap (*a* × *b* = 0.068, BCa 95% CI: 0.044, 0.092), self-efficacy (*a* × *b* = 0.030, BCa 95% CI: 0.009, 0.052), and resilience (*a* × *b* =0.022, BCa 95% CI: 0.001, 0.045) also significantly mediated the association between overcommitment and fatigue, respectively, and the mediating effect of self-efficacy and resilience accounted for 37.69 and 27.64%, respectively.

**Table 4:** Mediating roles of PsyCap's components.

**Mediators**	**EER**			**Overcommitment**		
	***a*_**1**_**	***b*_**1**_**	***a*_**1**_ × *b*_**1**_ (BCa 95% CI)**	***a*_**2**_**	***b*_**2**_**	***a*_**2**_ × *b*_**2**_ (BCa 95% CI)**
PsyCap	−0.280^**^	−0.379^**^	0.106 (0.078, 0.138)	−0.179^**^	−0.379^**^	0.068 (0.044, 0.092)
Self-efficacy	−0.225^**^	−0.203^**^	0.046 (0.016, 0.011)	−0.145^**^	−0.203^**^	0.030 (0.009, 0.052)
Resilience	−0.285^**^	−0.120^**^	0.034 (0.009, 0.070)	0.185^**^	−0.120^*^	0.022 (0.001, 0.045)
Hope	−0.243^**^	−0.080^*^	0.020 (−0.008, 0.050)	−0.152^**^	−0.080^*^	0.012 (−0.056, 0.033)
Optimism	−0.289^**^	−0.004	0.001 (−0.032, 0.034)	−0.182^**^	−0.004	0.001 (−0.020, 0.022)

* P < 0.05,

** *P < 0.01 (two-tailed). Age, gender, educational level, marital status, and shift patterns were adjusted. a_*1*_, association of EER with PsyCap's components; b_*1*_, association of PsyCap's components with fatigue; a_*1*_ × b_*1*_, the product of a_1_ and b_*1*_; BCa 95% CI, the bias-corrected and accelerated 95% confidence interval; a_*2*_, association of overcommitment with PsyCap's components; b_*2*_, association of PsyCap's components with fatigue; a_*2*_ × b_*2*_, the product of a_*2*_ and b_*2*_; BCa 95% CI, bias-corrected and accelerated 95% confidence interval*.

*EER, effort–reward ratio; PsyCap, psychological capital*.

## Discussion

This cross-sectional research investigated the level of fatigue among 1,104 physicians in Liaoning province, China. The result of the fatigue assessment in this study was 7.96 ± 3.95 (mean ± SD), and it was slightly higher than the score from medical personnel in Zhuhai (mean ± SD: 7.29 ± 3.24), a big city in China (3). Physicians in the study denoted higher levels of fatigue than did the scientific and technical personnel (mean ± SD: 7.28 ± 3.37) (50). And there was a significant difference in the level of fatigue on demographic characteristics such as age, educational level, and marital status. The level of fatigue among older physicians as well as the physicians with low levels of education needs to be taken seriously. The married and living together status seems to be positive factors in relieving fatigue, which possibly connected with family and friends support (25). In terms of working characteristics, physicians on shift reported a higher fatigue score than did physicians on fixed work. It is compatible with the result among nurses (11). Therefore, it can be recognized that rationalizing work pattern is an impactful measure for preventing fatigue.

After demographic and working characteristics were controlled for, occupational stress was significantly associated with fatigue. The correlation analysis indicated occupational stress was positively correlated with physicians' fatigue level, which means fatigue level rises as the imbalance between effort and reward becomes aggravated. Similarly, previous studies also showed that perceived stress was positively associated with fatigue (51, 52). One possible explanation for these findings is that physicians require comprehensive professional medical ability and sense of responsibility for patients, which makes them undertake a large number of learning tasks and work pressure even during nonworking hours. And high overcommitment leads to the high level of fatigue among physicians. The career promotion of Chinese physicians requires much scientific research outputs, which increases the difficulty and task in physicians' career advancement (53). They have spent too much time and energy on work, study, and scientific research; however, salaries and benefits of physicians still need to be improved (54). Finally, ERI resulted in fatigue among physicians. Therefore, hospital managers could establish a scientific performance appraisal and salary distribution system to balance the efforts and rewards, so as to reduce the occupational stress level and alleviate fatigue of physicians.

In addition, PsyCap was negatively correlated with Chinese physicians' fatigue, and it could mediate the association of occupational stress with fatigue. Similar to other studies, as a kind of positive psychological resource possessed by individuals, PsyCap is a protective factor to avoid or reduce turnover intentions, job dissatisfaction, and job burnout (23, 27). From previous studies, PsyCap has been considered as a mediator or moderator in some psychological, physical, and organizational relationships (48, 55, 56). Two components of PsyCap, self-efficacy and resilience, were both negatively associated with fatigue and could mediate the association between occupational stress and fatigue. It is worth noting that the proportion of the mediating effect of self-efficacy for ERR (17.20%) and overcommitment (37.69%) was respectively higher than the mediation rates of resilience for ERR (12.71%) and overcommitment (27.64%). A likely explanation is that self-efficacy represents an individual's confidence in ability to fulfill a task successfully (57). When physicians are faced with ERI and psychological and physical burden, low self-efficacy physicians may not perceive enough personal capabilities to perform successfully in complex undertakings (58). In other words, physicians with low occupational stress are more confident to do a good job and experience low levels of fatigue. Therefore, self-efficacy played a more important role in assuaging occupational stress and its effect on fatigue than resilience did. In terms of resilience, it can enable individuals to play a positive response to stressful environment and maintain physical and mental health (59). Chaukos found that building resilience can enhance concentration and coping skills and decline or prevent the aggravation of fatigue (60). Hence, a high level of resilience makes people able to recover from stressful experiences (like occupational stress) and buffers the effects of occupational stress on fatigue. Under the situation of ERI, it is feasible and effective to develop self-efficacy and resilience in the professional population like physicians for alleviating fatigue (61, 62).

Because PsyCap is developable, we suggest that the hospital administration can evaluate the PsyCap of physicians and implement targeted intervention. It is worth mentioning that Luthans et al. proposed PsyCap intervention (PCI) model (63), which provided guidelines in developing self-efficacy, resilience, hope, and optimism. In PCI training, we might develop self-efficacy by allowing physicians to experience success and accomplish their personal goals through peer encouragement; also, we could improve resilience by encouraging physicians to practice anticipating and addressing setbacks associated with the personal goals setting or with other events in work. Furthermore, bibliotherapy is a simple and inexpensive method that might be applicable to Chinese physicians, by organizing bibliotherapy workshops held in appropriate time in order to improve the PsyCap and relieve occupational stress and fatigue (64).

The Chinese version of the FS-14 has been used in our investigation, although Jing's paper indicated that the 11-item Chalder Fatigue Scale was superior than the FS-14 among the general population (65). But in our sampling population, the reliability tests showed that the internal consistency reliability of FS-14 was better than FS-11, and the confirmatory factor analysis confirmed that the validity of FS-14 is more satisfactory than that of FS-11. The possible explanation for these discrepant findings is that the fatigue performance of physicians is different from that of the general population.

There are several limitations in this research that must be revealed. Firstly, the cross-sectional design limited us to derive causal conclusions between variables studied. Further longitudinal studies are needed for extrapolating the causality. Secondly, participations were limited to the public tertiary hospitals in Liaoning, which did not represent all clinical workers in China; thus, extrapolating our results to physicians who work in other hospitals should be taken with caution.

## Conclusion

Chinese physicians have high levels of fatigue. Age, educational level, marital status, and shift patterns were significant indicators of fatigue, and occupational stress was positively associated with fatigue. Self-efficacy and resilience, two components of PsyCap, were negatively associated with fatigue and could mediate the association between occupational stress and fatigue. This finding offers the recommendation that individual positive psychological resources should be utilized and developed in physicians to reduce the high level of fatigue. Under a high level of occupational stress, development of PsyCap, self-efficacy, and resilience should be included in intervention strategies for minimizing fatigue targeted at Chinese physicians.

## Data Availability Statement

The raw data supporting the conclusions of this article will be made available by the authors, without undue reservation, to any qualified researcher.

## Ethics Statement

The survey was approved by the Provincial Department of Health and the Research Ethics Committee of the China Medical University. The patients/participants provided their written informed consent to participate in this study.

## Author Contributions

FT carried out investigation, data analysis and wrote the paper. QS provided help with the investigation and data collection. QC provided guidance in result interpretation. CL provided assistance in reviewing the paper. LW contributed toward investigation and data collection. HW provided guidance in study design, organized the investigation, and is the corresponding author. All authors approved the final manuscript.

### Conflict of Interest

The authors declare that the research was conducted in the absence of any commercial or financial relationships that could be construed as a potential conflict of interest.
